# Wearable Biosensing and Machine Learning for Data-Driven Training and Coaching Support

**DOI:** 10.3390/bios16020097

**Published:** 2026-02-04

**Authors:** Rubén Madrigal-Cerezo, Natalia Domínguez-Sanz, Alexandra Martín-Rodríguez

**Affiliations:** 1 Faculty of Education Sciences, UNIE Universidad, 28015 Madrid, Spain; ruben.madrigal@universidadunie.com; 2 Departamento de Ciencias de la Salud, Facultad de Ciencias de la Salud, Universidad Pública de Navarra (UPNA), 31500 Tudela, Navarra, Spain; natalia.dominguez@unavarra.es

**Keywords:** AI, machine learning, biosensing, wearable devices, adaptive training, digital coaching, human–AI collaboration, sports performance

## Abstract

**Background:** Artificial Intelligence (AI) and Machine Learning (ML) are increasingly integrated into sport and exercise through wearable biosensing systems that enable continuous monitoring and data-driven training adaptation. However, their practical value for coaching depends on the validity of biosensor data, the robustness of analytical models, and the conditions under which these systems have been empirically evaluated. **Methods:** A structured narrative review was conducted using Scopus, PubMed, Web of Science, and Google Scholar (2010–2026), synthesising empirical and applied evidence on wearable biosensing, signal processing, and ML-based adaptive training systems. To enhance transparency, an evidence map of core empirical studies was constructed, summarising sensing modalities, cohort sizes, experimental settings (laboratory vs. field), model types, evaluation protocols, and key outcomes. **Results:** Evidence from field and laboratory studies indicates that wearable biosensors can reliably capture physiological (e.g., heart rate variability), biomechanical (e.g., inertial and electromyographic signals), and biochemical (e.g., sweat lactate and electrolytes) markers relevant to training load, fatigue, and recovery, provided that signal quality control and calibration procedures are applied. ML models trained on these data can support training adaptation and recovery estimation, with improved performance over traditional workload metrics in endurance, strength, and team-sport contexts when evaluated using athlete-wise or longitudinal validation schemes. Nevertheless, the evidence map also highlights recurring limitations, including sensitivity to motion artefacts, inter-session variability, distribution shift between laboratory and field settings, and overconfident predictions when contextual or psychosocial inputs are absent. **Conclusions:** Current empirical evidence supports the use of AI-driven biosensor systems as decision-support tools for monitoring and adaptive training, but not as autonomous coaching agents. Their effectiveness is bounded by sensor reliability, appropriate validation protocols, and human oversight. The most defensible model emerging from the evidence is human–AI collaboration, in which ML enhances precision and consistency in data interpretation, while coaches retain responsibility for contextual judgement, ethical decision-making, and athlete-centred care.

## 1. Introduction

Over the past three decades, coaching has shifted from an intuitive craft to a recognized professional practice used across sport, organizations, education and healthcare to support learning, performance and personal growth [1,2]. Coaching is typically conceptualized as a collaborative, goal-focused and reflective relationship in which a coach uses techniques such as open questions, active listening and structured feedback to help clients pursue self-valued goals and enhance self-awareness and self-regulation [2]. Relational and motivational models emphasise that the quality of the working alliance is one of the strongest predictors of outcomes in both sport and organisational coaching [3], and research in strength and conditioning (S&C) highlights trust, respect, clear communication, individualised attention and holistic care for the “whole person” as core ingredients of effective coach–athlete relationships [4].

While this relational tradition has consolidated, the last 15–20 years have seen a rapid expansion of technologies that quantify human movement, physiology and behaviour in sport, health and everyday life [5,6]. In sport, GPS, inertial measurement units (IMUs), force plates, heart-rate monitors and digital questionnaires have turned training and competition into data-rich processes, while Machine Learning (ML), neural networks and deep learning (DL) are increasingly used to analyse complex movement patterns, estimate joint and tissue loads, predict injury risk and individualise training based on large multimodal datasets [5,6]. Narrative reviews of Artificial Intelligence (AI) in sport describe a rapidly expanding ecosystem of tools for performance analysis, talent identification, injury prevention, sports medicine and health monitoring [5]. Thus, from an engineering and systems perspective, contemporary AI-driven coaching architectures are increasingly structured around wearable biosensing pipelines. Within this framework, raw physiological, biomechanical, and biochemical signals constitute the sensing layer, which must be supported by robust signal conditioning and quality control procedures to address noise, artefacts, sensor drift, and contextual variability inherent to real-world exercise environments [5,6]. These preprocessed signals are subsequently transformed through feature extraction and representation learning, enabling ML models to perform inference and generate training prescriptions. Recent research emphasizes the importance of uncertainty-aware decision-making, validation boundaries, and human oversight to prevent overconfident or maladaptive recommendations when data quality or contextual information is limited. Continuous feedback and monitoring allow systems to update predictions and prescriptions dynamically as new sensor data become available. This layered architecture highlights that higher-level adaptive coaching functions remain fundamentally constrained by the validity, reliability, and interpretability of the underlying biosensor pipeline [6].

In other contexts, AI is reshaping medical education, rehabilitation and lifestyle medicine. In medical and health professions education, expert systems, intelligent tutoring systems and AI-enhanced simulations support the teaching of theoretical knowledge, procedural skills and clinical reasoning, with virtual patients and virtual instructors sometimes matching or exceeding human instructors in narrow technical domains such as neurosurgical simulation [7,8]. Lifestyle medicine (LM) focuses on preventing, managing and often reversing chronic noncommunicable diseases through changes in nutrition, physical activity, sleep, stress, substance use and social connection [9], and AI enables precision lifestyle medicine by integrating data from wearables, mobile apps, electronic health records (EHRs), genomics and social determinants to generate personalised, dynamically updated prescriptions supported by virtual coaches and cognitive-behavioural chatbots [9]. In education and public health, physical-education (PE) research documents the integration of wearables, fitness apps, exergames, augmented and virtual reality (AR/VR) and AI-based assessment systems to enhance engagement, support motor learning, extend activity beyond class time and encourage lifelong physical activity, while also responding to rising childhood overweight and obesity despite compulsory PE curricula [10].

Concurrently, digital health and telemedicine are moving healthcare from episodic, hospital-centred encounters toward distributed ecosystems in which patients are monitored via wearable sensors, remote patient-monitoring (RPM) platforms and the Internet of Medical Things (IoMT), and where AI supports early disease detection, prognostic modelling and precision treatment [11]. These developments promise more preventive, personalized care and greater patient empowerment, but also raise concerns about accessibility, equity, privacy, security, bias and governance, especially in low- and middle-income countries with limited digital infrastructure and regulatory capacity [11].

In parallel, digital coaching has emerged as a rapidly growing field: video-, phone- and chat-based coaching became widespread during the COVID-19 pandemic, and global providers such as BetterUp, CoachHub and EZRA have attracted substantial venture-capital investment [2]. Definitions remain contested—some restrict “digital coaching” to synchronous audio- or video-based conversations between human coach and coachee, whereas others extend the term to AI-based coaching, digital-training apps and wearable-driven feedback systems—and at least one recent trial suggests that AI-based coaching can, under certain conditions, achieve goal-attainment outcomes comparable to human coaches [2,12].

At the same time, a growing body of critical scholarship on AI wearables, digital health and algorithmic decision-making highlights profound ethical, legal and governance challenges. Passive and pervasive monitoring, opaque “black-box” models and biased datasets can threaten privacy, autonomy, fairness and trust [13], and regulatory frameworks such as GDPR, emerging AI acts and medical-device regulations increasingly demand transparency, explainable AI, bias mitigation and multi-level accountability for AI-enabled health systems [11,13].

Against this backdrop, the central question of this article must be addressed within an ecosystem where coaching is relational and motivational, data are increasingly granular and continuous, and AI systems range from analytic engines and diagnostic aids to conversational agents, digital twins and semi-autonomous recommendation systems [4,5,9,14]. Rather than treating “AI” and “the coach” as monolithic entities, we synthesise evidence from sport, biomechanics, biosensing, education, lifestyle medicine, digital health and coaching research to describe how coaching is evolving in the age of data, and to identify which components of coaching are most amenable to automation and which remain fundamentally human. Within this framework, sensing, signal quality, feature representation, inference, and feedback constitute the technical backbone, while higher-level applications remain constrained by the validity, reliability, and safety of the underlying sensor data [5,6,11].

### Methodology

This review employed a structured narrative synthesis approach, aiming to integrate both empirical evidence and contextual literature relevant to AI, biosensing technologies, and data-driven coaching systems. Given the conceptual breadth of the topic, the heterogeneity of technological applications, and the rapid evolution of AI-driven tools, a narrative framework was selected to facilitate the inclusion of studies that provide theoretical, methodological, and applied context alongside empirical findings.

A comprehensive literature search was conducted across PubMed, Scopus, and Web of Science, and complemented by targeted searches in Google Scholar, covering publications from 2010 to 2026. Search strategies combined controlled vocabulary and free-text terms using Boolean operators (OR/AND), adapted to the syntax of each database. Search strings were organised into conceptual blocks addressing: AI (*“Artificial Intelligence” OR “machine learning”*); digital and adaptive coaching (*“digital coaching” OR “virtual coaching” OR “adaptive training”*); biosensing and monitoring technologies (*“biosensing” OR “wearables” OR “digital twins”*); and sport-related contexts (*“sport performance”*). Reference lists of key articles and recent reviews were additionally screened to identify relevant studies not captured through the primary search.

This process yielded a total of 826 records identified across databases and targeted searches (PubMed, n = 193; Scopus, n = 112; Web of Science, n = 96; Google Scholar, n = 425). After merging records and removing duplicates and clearly ineligible entries based on title and abstract screening (n = 566), 260 records remained for further assessment.

Titles and abstracts were screened for relevance, leading to the exclusion of 72 records that did not meet the scope of the review. Eligibility criteria included: (a) empirical studies, systematic reviews, scoping reviews, technological reports, or conceptual papers; (b) a focus on AI-assisted training, biosensing technologies, digital coaching systems, or automated decision-making in sport, physical activity, or exercise-related contexts; and (c) publications in English. Studies were excluded if they lacked sufficient methodological transparency or focused exclusively on non-human, clinical, or industrial applications without relevance to coaching or training.

**Figure 1:**
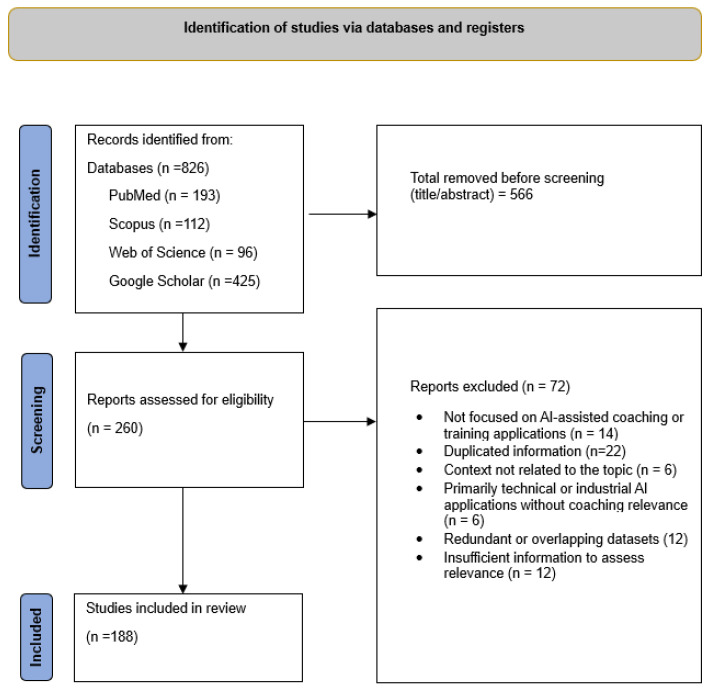
PRISMA 2020 flow diagram illustrating the identification, screening, eligibility assessment, and inclusion of studies in this structured narrative review on AI–driven biosensing and data-driven coaching systems.

The remaining 188 studies were included in the narrative synthesis. Each study was retained based on its contribution to understanding the technological capabilities, limitations, and practical implications of AI-driven coaching systems. Given the diversity of study designs and objectives, methodological rigor, ecological validity, and technological maturity were evaluated narratively. The study selection process is summarized in a PRISMA 2020 flow diagram (Figure 1).

Accordingly, this narrative review aims to examine whether AI can meaningfully replicate, complement, or replace selected functions of the human coach in training design, monitoring, and decision-making. By synthesising evidence across biosensing technologies, machine learning–driven adaptive systems, digital coaching tools, and emerging immersive solutions, this review delineates current capabilities, limitations, and ethical implications of AI-driven coaching, while clarifying the domains in which human expertise remains indispensable. For clarity, AI is used as an umbrella term referring to computational systems capable of inference, adaptation, or decision support. Within this scope, Machine Learning (ML) and deep learning (DL) denote data-driven statistical and neural approaches applied to biosensor-derived signals for prediction, classification, and training adaptation. In contrast, when referring to AI-based interaction or communication systems (e.g., large language models used for feedback, guidance, or dialogue with athletes), this is explicitly stated and treated as a distinct application layer, rather than as the core analytical engine of the adaptive training system. Also, “coaching” is addressed from the perspective of data-driven training monitoring and decision support, rather than encompassing the full spectrum of tactical, regulatory, or pedagogical aspects traditionally associated with human coaching expertise.

Table 1 highlights sensor modalities, validation settings, model types, and evaluation protocols, illustrating both the potential and the methodological constraints of AI-driven adaptive training systems.

## 2. From Relational Coaching to Sensor-Based Training Ecosystems

### 2.1. From Experiential Coaching to Early Quantification

Historically, coaching in sport was largely experiential and intuition-driven: coaches relied on direct observation, personal playing or leadership experience and informal apprenticeship to judge effort, technique and readiness, with limited systematic measurement of psychological or physical variables [4]. Early psychological models, such as Mageau and Vallerand’s motivational sequence, began to formalise this craft by specifying how coaches’ interpersonal orientations and contextual pressures shape autonomy-supportive versus controlling behaviours, athletes’ basic psychological needs and the quality of motivation and persistence, introducing measurable constructs—coaching behaviours, need satisfaction, motivation type, persistence and dropout—that could be quantified rather than inferred solely from experience [3,14].

In strength and conditioning (S&C), this movement toward quantification extended into the relational domain, and research on leadership styles, leadership perceptions, coach knowledge and relationship-building uses leadership scales, coaching-behaviour questionnaires and perception measures to link democratic versus autocratic styles and training/instructional and social-support behaviours with athlete outcomes [4,31,32].

Similar trends appeared in lifestyle medicine, physical-activity promotion and PE, where traditional interventions based on brief counselling, paper diaries, pedometers and SMS reminders produced only coarse, episodic data, and early step counters, basic fitness apps and rule-based prompts introduced a first, generic quantification of activity, sleep and sometimes diet that was only partially integrated into daily life [9].

In PE, technology moved from simple ICT tools to step-tracking apps, video replay and basic heart-rate sensors, allowing distance, steps, pace or heart rate to be quantified in class projects, while the COVID-19 pandemic accelerated online and hybrid formats, revealing both the possibilities of data-enabled teaching and persistent infrastructure gaps [10]. In early digital health, wireless sensor networks and remote-monitoring systems extended clinical oversight beyond episodic visits by providing continuous, though still coarse, vital-sign data, and accelerometer-based datasets such as MobiAct, MobiFall, UniMiB SHAR and UCI HAR were used with classical ML algorithms to recognise basic activities and detect anomalies, marking a first generation of AI-assisted quantification of everyday behaviour and a broader shift from purely observational practice to explicit measurement and analysis of psychological constructs, behaviours and physical outputs, which laid the foundations for contemporary AI-driven systems based on richer, continuous data streams in sport and health [11,13].

### 2.2. Wearable Technology and the Quantified Athlete

The proliferation of wearable technology has transformed monitoring of training load, recovery and health, with commercial devices providing continuous data on heart rate, movement and sleep in both elite and recreational contexts [5,6]. New biosensing systems based on flexible patches, textiles and advanced nanomaterials extend this to molecular information from sweat and other fluids, offering markers of hydration, metabolic intensity, stress and endocrine function [6,33]. The same tools underpin the “quantified self” and precision lifestyle medicine, support more active lifestyles in PE and youth sport, and feed vital-sign data into telemedicine and IoMT infrastructures for remote care [9,11]. However, variable accuracy, motion artefacts, demographic bias, missing data and unresolved privacy and regulatory issues still constrain the use of wearables in high-stakes decisions about training, health or return-to-play [13].

### 2.3. From Monitoring to Decision Support and Machine Learning

As wearable and biosensing data streams have multiplied, AI and ML have become central to turning raw signals into decision-relevant information: in sport, models applied to kinematic, kinetic, EMG, workload and tracking data classify movement, detect technical errors, estimate joint and tissue loads and link spatiotemporal patterns to injury risk and game outcomes, while in sports medicine, rehabilitation and lifestyle medicine they support injury detection, prognosis, return-to-sport decisions and prediction of chronic-disease trajectories, and underpin virtual coaches, CBT-based chatbots and digital sleep programmes that deliver just-in-time adaptive interventions [5,6,9,34]. Methodologically, these systems share pipelines of pre-processing, feature extraction and supervised/unsupervised learning plus, increasingly, reinforcement learning and edge computing for closed-loop, low-latency adaptation [6,11,13,33]

### 2.4. Digital and AI-Enhanced Coaching Platforms

The convergence of sensing, analytics and user interfaces has produced digital platforms that act as AI-enhanced coaching environments, integrating multimodal data capture, AI-based analytics and user-facing feedback across sport, health and education [11]. In sport, athlete-management and tactical-analysis systems combine wearable and tracking data with dashboards and simple ML-based alerts to inform decisions on load, readiness and game strategy, while LMS-based tools help coaches interpret biomechanical analyses and refine technical feedback 5. In healthcare and lifestyle medicine, telemedicine, RPM platforms and AI-powered virtual coaches use sensor and wearable data to support remote monitoring and dynamically updated exercise, nutrition, sleep and stress-management plans [9]. In PE and youth sport, exergames, gamified apps and AR/VR experiences function as digital coaches that adapt difficulty, provide real-time feedback and increase motivation and activity, and emerging digital-twin approaches hint at future systems that simulate individual responses to interventions to guide adaptive training and rehabilitation planning [10].

### 2.5. Reframing the Role and Value of the Human Coach

As data-rich technologies and AI-enhanced platforms proliferate, the role of the human coach or health professional is shifting from being a primary source of technical information to acting as an architect of motivational climates and working alliances that sustain engagement and adaptation, with autonomy-supportive, empathic and holistic behaviours remaining central for motivation, adherence and performance [3,4,9,14,31]. Evidence across sport, health and education points to a clear complementarity: AI excels at processing large data volumes, identifying patterns and quantifying risk, while human experts are indispensable for integrating these outputs with values, preferences, context and relationships, and for managing ethical issues around privacy, autonomy, bias and the potential misuse of sensitive data [5,7,9,11,13]. Accordingly, the rise of AI literacy as a curricular priority reflects an expectation that coaches, clinicians and teachers become informed, critical co-designers and users of AI tools who can interpret and challenge algorithmic outputs and act as ethical guardians and integrators of AI-generated insights within rich, human relationships, rather than as professionals to be replaced by algorithms [4,7,8,35].

## 3. Biosensing as the Foundational Layer of AI-Driven Training Systems

Biosensing technologies constitute the fundamental infrastructure of any artificial coaching system. By continuously monitoring physiological, biochemical, and biomechanical responses, they generate the information that enables AI models to understand, predict, and adapt to the human body’s reactions to training [36]. The transformation of subjective observation into quantifiable biosignal data has changed how performance and adaptation are interpreted, allowing the emergence of data-driven coaching systems capable of real-time feedback and decision-making. From a systems-engineering perspective, wearable biosensing represents the foundational layer of closed-loop adaptive training systems. All subsequent processes, including signal conditioning, feature extraction, MLinference, and adaptive prescription, are strictly dependent on the quality, resolution, and contextual validity of the sensed biological signals. Limitations at the sensing level, therefore, propagate downstream, defining the upper bounds of model performance, decision reliability, and training safety [6,13,33].

### 3.1. From Physiological to Biochemical and Biomechanical Sensing

Modern wearable technologies allow the simultaneous acquisition of multiple biological signals. Physiological monitoring, including heart rate, heart rate variability, oxygen saturation, respiration, or skin temperature, provides a direct window into internal load and recovery [37,38]. More advanced sensors, such as electromyography (EMG) or electroencephalography (EEG), capture neuromuscular and cognitive states during exercise, contributing to a deeper understanding of effort perception and motor control [39]. Recent controlled studies have confirmed the validity of these indicators: for example, Santos-García et al. (2022) demonstrated that HRV reliably reflects internal load fluctuations in elite female soccer players during match weeks [15], while de Beukelaar et al. (2023) found strong correlations between HRV indices and training intensity and resistance training [40]. Longitudinal evidence also supports these associations, as O’Connor et al. (2024) observed consistent relationships between waking HRV and training load over two competitive seasons in professional Australian Rules Football players [16]. More advanced sensing approaches, such as electromyography (EMG) and electroencephalography (EEG), are increasingly used to assess neuromuscular activation and cognitive workload during exercise, contributing to a deeper understanding of motor control and fatigue mechanisms [17].

Alongside these systems, biochemical biosensors now offer noninvasive access to metabolic and hormonal information through sweat, saliva, or interstitial fluid [18]. Miniaturized microfluidic and electrochemical devices can detect key biomarkers such as glucose, lactate, cortisol, and electrolytes, helping to identify stress levels, dehydration, or metabolic fatigue. Yang et al. (2024) developed a wearable device capable of continuously monitoring lactate concentration in sweat during endurance exercise, reporting strong correlations (r > 0.85) with blood lactate levels across incremental workloads [19]. Similarly, Luo et al. (2025) validated a flexible microfluidic patch that simultaneously measured sodium and potassium concentrations in sweat, which reflected real-time electrolyte dynamics and metabolic stress in trained cyclists. Salivary biosensors have also emerged as promising tools [18]. Swetha et al. (2022) pointed out in a minireview that there is an electrochemical sensor that detects cortisol fluctuations associated with exercise-induced stress, enabling noninvasive hormonal tracking [20]. These findings are consistent with broader results emphasizing that next-generation biochemical wearables can provide reliable insights into metabolic state, hydration, and recovery without requiring invasive sampling procedures [41]. These biochemical parameters, once only measurable in laboratory settings, are increasingly integrated into wearable platforms that operate in real-world training environments.

Biomechanical sensors, including accelerometers, gyroscopes, and inertial measurement units, add a complementary dimension to physiological and biochemical data. They capture motion quality, coordination, and efficiency, allowing AI systems to detect deviations in technique or mechanical fatigue patterns that might increase injury risk [42]. When combined, these multimodal biosignals provide a dynamic and multidimensional view of the athlete that extends beyond human observation [21].

### 3.2. From Data Streams to Intelligent Interpretation

The transition from raw biosignals to actionable intelligence requires robust signal conditioning, quality control, and data fusion mechanisms. Noise, motion artefacts, sensor drift, and contextual variability, particularly in field-based monitoring, must be addressed before any meaningful feature representation or model inference can occur. This intermediate layer is critical to preventing error amplification and ensuring the physiological and biomechanical validity of AI-driven decisions [22,23,43,44,45]. The power of biosensing depends not only on measurement precision but on the capacity to transform continuous data streams into meaningful insights. Raw signals are often affected by noise, drift, or contextual variability, especially in field conditions [43]. To overcome these limitations, AI-driven signal processing and data fusion frameworks, such as deep learning autoencoders or Kalman filters [22,23], are increasingly applied to refine, synchronize, and interpret complex inputs [44,45].

Recent methodological advances in AI have significantly improved the processing and interpretation of biosensor data, enhancing precision and real-time adaptability in training environments. Zhang et al. (2025) proposed a hybrid approach combining a Kalman filter with a Kernelized Correlation Filter (KCF) to reconstruct complex motion trajectories, demonstrating that this integration effectively reduces signal noise and improves motion tracking accuracy under dynamic conditions, principles that can be directly applied to biomechanical monitoring in athletes [22]. Similarly, Wen and Diliya utilized a Variational Autoencoder (VAE) model to extract latent features from multisensor datasets, enabling personalized training recommendations by capturing individual response patterns and adaptation trends [23]. In parallel, Zunino et al. illustrated how AI-driven diagnostic models can detect subtle deviations in sensor signals, originally within industrial systems, but with clear parallels to identifying early fatigue or performance degradation in physiological data [45]. Complementing these approaches, recent analyses on real-time stress detection emphasize the integration of Internet of Things (IoT) frameworks with AI to continuously monitor and modulate stress responses, suggesting a future where wearable systems autonomously adapt exercise intensity based on psychophysiological feedback [44].

The integration of physiological, biochemical, and biomechanical data enables algorithms to recognize hidden patterns of adaptation, anticipate fatigue, or adjust workloads automatically. In this sense, biosensing forms the perceptual system of the artificial coach: it senses and translates the biological reality of the athlete into interpretable signals that guide decision-making. As wearable technologies continue to evolve toward higher fidelity, flexibility, and comfort, their symbiosis with AI brings us closer to an intelligent coaching paradigm capable of mirroring the responsiveness and contextual awareness of expert human trainers [46].

The practical impact of biosensing integration in training has also been supported by controlled experimental evidence. In a randomized controlled trial, Wang et al. (2025) showed that athletes who trained with continuous wearable-based feedback for twelve weeks exhibited significantly improved adaptation markers compared to a control group without sensor guidance, including greater heart rate variability recovery, reduced perceived fatigue, and enhanced performance stability [24]. Similarly, Lundstrom et al. (2024) demonstrated that wearable-derived physiological metrics, particularly HRV and oxygen consumption estimates, correlated strongly with measured training load (r = 0.70–0.80) and energy expenditure, confirming that biosensor data can accurately reflect internal and external workload dynamics [25]. Together, these findings provide experimental validation that real-time, multimodal biosensing enhances the precision of training monitoring and supports adaptive responses that align with the objectives of intelligent coaching systems (Table 2).

#### Operational Signal Processing, Quality Control, and Multimodal Integration Considerations

Preprocessing gains should be evaluated by reporting performance and calibration metrics before and after the preprocessing/QC pipeline under identical data splits, and by stress-testing robustness under simulated missingness (channel dropout) and domain shift (cross-device or cross-setting validation). To facilitate operationalization and reproducibility, Table 3 summarizes recommended preprocessing, quality-control, synchronization, and windowing practices across common wearable sensing modalities used in exercise monitoring.

Additionally, multimodal wearable data are commonly integrated using early, late, or hybrid fusion strategies, each presenting distinct trade-offs relevant to real-world deployment. Early fusion concatenates features from multiple sensors prior to model training, enabling the capture of cross-modal interactions but at the cost of increased sensitivity to time misalignment, feature scaling, and missing channels, as well as higher computational demands and limited explainability. Late fusion combines modality-specific model outputs, offering improved robustness to sensor dropout, lower computational cost, and greater interpretability, albeit with reduced capacity to model fine-grained cross-sensor dependencies. Hybrid fusion architectures, which combine modality-specific encoders with intermediate fusion layers (e.g., attention mechanisms), balance interaction modeling and robustness but introduce additional complexity and risk of overfitting in small or heterogeneous datasets. Regardless of strategy, robustness and generalization should be explicitly evaluated through ablation studies under simulated channel dropout, cross-device or cross-setting domain shift, and calibration analyses to assess confidence reliability when sensor availability or data quality varies.

### 3.3. Precision, Calibration, and Failure Modes in Field Conditions

In field conditions, as specified by Su et al. and specifically in muscle activity by Skalski et al., wearable biosensing performance is primarily constrained by modality-specific failure modes that directly propagate into model training and deployment [47,48]. For wrist and limb PPG, motion artifacts and contact-force variability can dominate the waveform, while peripheral perfusion changes driven by temperature, vasoconstriction, and local vascular dynamics can reduce signal amplitude and distort morphology, motivating the use of signal-quality indices and accelerometer-informed gating to discard low-quality windows or down-weight corrupted segments [49]. For surface EMG, electrode–skin impedance variability, sweat-related changes in contact, and electrode placement repeatability are major contributors to inter-session variability, requiring explicit normalization (e.g., MVC-based amplitude normalization) and standardized protocols to enable comparability across sessions, participants, and contexts [50,51,52]. Wearable sweat sensing introduces additional sources of error due to cross-sensitivities (e.g., pH, temperature, interfering analytes), along with sweat-rate dependence and environmental effects that necessitate calibration/compensation and QC thresholds before downstream inference [53,54]. IMU-based monitoring is affected by sensor misalignment, drift, and context-dependent movement patterns, which can yield dataset shift between laboratory and field settings; accordingly, QC flags for missing/implausible data and evaluation schemes that test subject- and context-independence are critical to avoid inflated performance estimates and false confidence at deployment [55]. Across modalities, these artefacts and calibration requirements are not merely signal-processing concerns: they define the effective data-generating process and therefore shape model generalization, uncertainty calibration, and the reliability of any training prescription derived from sensor-driven inference.

## 4. Machine Learning for Biosensor-Based Adaptive Training

The application of ML techniques has transformed how exercise data are interpreted and applied to guide training decisions. By leveraging large, multimodal datasets from biosensors, ML algorithms can identify patterns that are not perceptible to human analysis, allowing for individualized training prescriptions that evolve dynamically with the athlete’s physiological state [56]. Supervised learning models, such as decision trees, support vector machines, and neural networks, are commonly used to predict outcomes like performance level, recovery time, or injury risk based on historical and real-time inputs. These systems enable the creation of adaptive feedback loops in which training variables intensity, duration, and recovery are continuously optimized according to the athlete’s response [57]. Thus, ML operates downstream of biosensing and signal processing, transforming structured features into predictive and prescriptive outputs. The reliability and generalizability of model inference are therefore intrinsically bounded by sensor accuracy, feature robustness, and validation protocols. ML should thus be interpreted as an analytical core, not an autonomous decision-maker, embedded within a broader human-supervised adaptive training system [6,13,47].

Recent studies have demonstrated the practical potential of ML applications in sports performance and training adaptation. Mohapatra et al. (2024) developed a multilevel fatigue prediction framework using wearable inertial sensors, heart rate, and motion data, showing that their model could accurately classify physical fatigue states and predict onset during endurance exercise [58]. Similarly, Aranda et al. (2025) employed supervised and deep learning algorithms trained on physiological and GPS data from endurance athletes to predict recovery and fatigue status, achieving higher predictive accuracy than conventional workload-based methods [59]. Beyond fatigue monitoring, Dindorf et al. (2025) highlighted the versatility of MLin biomechanical analysis, emphasizing that both supervised and unsupervised approaches such as clustering and dimensionality reduction can detect subtle kinematic variations and inform individualized training adjustments [60]. Collectively, these findings support the growing integration of AI and biosensor data for the design of adaptive, responsive, and data-driven training systems.

### 4.1. ML in Endurance Training

Endurance sports have served as a fertile ground for applying ML to monitor fatigue, recovery, and performance fluctuations. Rothschild et al. (2024) conducted a 12-week controlled study in 43 endurance athletes, showing that ML models could accurately predict next-day recovery status and morning heart rate variability, achieving lower error rates (RMSE = 11.8) compared with traditional recovery models (RMSE = 14.1) [26]. Similarly, Qin et al. (2025) developed a hybrid deep learning framework combining physiological (HRV, VO_2_, EMG) and psychological data, reaching an explained variance of R^2^ = 0.90 in predicting athletic performance across 480 participants [27]. These results confirm that integrating multimodal biosensor inputs enhances model precision for predicting adaptive responses and optimizing endurance training.

Complementary findings by Wang C. (2024) demonstrated that combining recognition algorithms with physiological fatigue assessment could optimize training load and improve efficiency, suggesting that ML systems can autonomously balance performance gains with recovery needs [61]. Collectively, these studies show that ML-based models in endurance contexts can dynamically tailor exercise prescriptions, outperforming static approaches in predicting physiological readiness and fatigue onset.

### 4.2. ML in Strength, Skill, and Multimodal Training

In contrast to endurance-based applications, other sports domains—such as resistance, team, and skill-based training—have increasingly adopted ML for biomechanical and neuromuscular optimization. Guneralp et al. (2025) used four ML models, including decision trees, random forest, and XGBoost, to classify athletes’ adaptive responses to strength and small-sided football training sessions. The XGBoost model achieved the highest predictive accuracy (0.73–1.00), effectively differentiating between adaptation patterns to combined and isolated training modalities [28]. Such approaches reveal how ML can identify performance trends that are invisible to conventional analytics, guiding coaches in personalizing exercise design.

Moreover, these findings align with growing evidence that ML-driven biomechanical modeling can detect subtle kinematic or electromyographic variations associated with fatigue, asymmetry, or skill degradation. By integrating these insights into feedback systems, ML-based platforms can provide real-time guidance for improving motor control and technical execution [21,62]. This demonstrates the capacity of AI to bridge physical performance data with adaptive coaching decisions in strength and team-sport environments.

Overall, ML serves as the analytical core of the artificial coach, converting raw biosensor data into actionable intelligence. Through predictive modeling and adaptive learning, these systems can tailor training stimuli to optimize performance while minimizing fatigue and injury risk. As algorithmic transparency and computational efficiency improve, ML-driven training design is moving closer to real-time implementation, enabling intelligent, continuously learning exercise environments that complement and extend human coaching expertise.

### 4.3. Minimum Reporting Checklist for ML-Enabled Adaptive Training Systems

Given the increasing use of ML models to inform training adaptation, recovery management, and injury-risk estimation, the credibility and safety of such systems depend not only on predictive performance but also on transparent and appropriate evaluation practices. To support reproducibility and responsible deployment, we outline below a minimum reporting checklist for ML-enabled adaptive training systems:

- Training, validation, and testing splits should be performed at the athlete level to prevent information leakage, and time-forward validation schemes should be used when models are intended for prospective decision support [63].
- Performance should be contextualized against appropriate baselines, including simple statistical models and ablation analyses (e.g., single-modality models or no-fusion baselines) to quantify the added value of model complexity [64].
- In addition to accuracy-based metrics, calibration measures (e.g., expected calibration error, Brier score) should be reported, and uncertainty estimates should be used to define confidence thresholds or human-in-the-loop triggers when predictions are unreliable [65].
- Where external validation is not feasible, models should be evaluated under distribution shift scenarios (e.g., season-to-season, team-to-team, or sport-to-sport transfer) to assess robustness beyond the development dataset [66].
- Interpretability should be aligned with the decision context, enabling practitioners to understand which signals and conditions drive model outputs, rather than relying solely on post hoc explanations detached from the training prescription process [67].

### 4.4. Synthesis and Implications

Across both endurance and non-endurance contexts, ML (ML) serves as the analytical core of the artificial coach. By transforming biosensor-derived information into predictive and prescriptive intelligence, ML transcends the role of a mere analytical tool and begins to function as a virtual coach [68,69], one capable of learning from experience, adjusting its decisions, and providing individualized guidance in real time. Through predictive and adaptive modeling, ML can anticipate fatigue, optimize recovery windows, and minimize injury risk by continually refining its understanding of each athlete’s physiological and behavioral responses.

As algorithmic transparency, computational power, and sensor integration continue to evolve, these systems are becoming increasingly autonomous and interactive, capable of simulating some aspects of human coaching judgment [29]. They can detect deviations in performance, suggest optimal workloads, and adapt training prescriptions instantaneously in response to new data streams. This transformation blurs the boundary between monitoring and decision-making, positioning ML not just as a diagnostic framework but as an intelligent agent capable of participating in the coaching process itself. However, such autonomy can also have unintended consequences. For instance, a recent pilot by Silacci et al. demonstrated that an AI-driven workload adjustment system in cyclists occasionally overestimated recovery readiness when sleep quality or psychological stress indicators were absent from the model, leading to premature increases in training intensity and transient performance decline [29]. This example underscores the persistent challenge of contextual awareness in machine learning, despite its analytical precision.

Despite these promising advances, the concept of MLas a coach also raises several concerns and limitations. Current algorithms, while powerful in pattern recognition, still lack contextual awareness, empathy, and the ability to integrate psychosocial factors that often determine adherence and motivation in training. As Naughton et al. (2024) emphasize, excessive reliance on automated decision systems in sport and health contexts may lead to over-optimization, data fatigue, or misinterpretation of physiological signals when environmental and psychological variability are not adequately modeled [70]. Furthermore, issues of data privacy, algorithmic bias, and interpretability remain unresolved, challenging the ethical deployment of AI-based systems that influence human performance and well-being.

## 5. Closed-Loop Adaptive Training: Capabilities and Limits of the Artificial Coach

The development of intelligent systems for training and physical exercise has advanced at an exponential rate in recent years, particularly in end-user products aimed at the general adult population. In a society increasingly shaped by immediacy, it is essential to understand the *real* capabilities and limitations of these systems. The artificial coach can be conceptualized as the decision and prescription layer of a closed-loop adaptive training system. Its capabilities emerge from the interaction between biosensing, ML inference, uncertainty management, and feedback mechanisms. Accordingly, the limitations of artificial coaches are not only algorithmic but also originate from sensor variability, incomplete contextual inputs, and constraints in validation and safety envelopes [5,11,13].

### 5.1. What Do We Mean by “Artificial Coach”?

From early clinical applications in the 1960s—such as blood glucose determination [71] and earlier milestones such as the mercury thermometer, measurement through biosensors has progressed substantially. The key is to understand what is being measured and why such information is being collected. Measurement generates data outputs that are translated into prescriptions, alerts, feedback, and similar actions. In training contexts, these outputs have evolved and have progressively been integrated into coaching practice. Below, we outline the device and technology typologies that currently shape what an “artificial coach” may mean.

According to Zhou et al. (2025), several typologies can be distinguished within the artificial coach concept, including training apps with fixed rules, recommendation systems and wearable devices, chatbots, multi-coach systems, and digital twins. Digital twins combine 3D modeling, the Internet of Things (IoT), and AI (hereafter, AI) to replicate a physical object or system. This can support the monitoring of physiological and biomechanical variables, enabling optimization of technical–tactical or physical performance, as well as injury prevention and rehabilitation.

Fixed-rule apps typically propose simple weekly training schedules without requiring biosensors for deployment [5,72]. Chatbots, depending on the user’s objective and the system’s programming, may provide responses based either on broader reasoning and analysis of data available online or on simpler word-sequencing approaches derived from training and algorithmic design, to support goals such as performance, training, or nutrition. A central limitation of chatbots or AI generated through “LLM-coaches” is that the internal process producing outputs (the “black box”) is not publicly transparent [5].

Recommendation systems and wearable devices provide users with suggestions about what to do and in what quantity, often as a first “intelligent layer” supported by biosensors [73]. Artificial coach systems can go a step further by adjusting intensity, positive reinforcement, or training frequency, whereas multi-component systems combine these elements and may include human oversight, more sophisticated algorithms, and, in some cases, educational content. Some systems aim to create effective and motivating training through continuous monitoring [74].

Overall, these technologies can quantify physiological, biomechanical, positional/location-based, or environmental parameters [75]. Based on data acquisition, an output is delivered as feedback. This feedback is then interpreted by the person managing the devices, who processes the information and acts accordingly, depending on the criteria used to categorize and apply the data.

A systematic review by Rebelo et al. concluded that data provide relevant information about the player, enabling load personalization according to sport-specific demands, supporting injury recovery, and addressing positional or physical requirements of the activity [76]. In this way, an artificial coach can support training management for the human coach. This, essentially, is the meaning of “artificial coach” nowadays.

An artificial coach is not a substitute for a human coach. Without adequate training to interpret device-generated data, health consequences can be dire. The artificial coach should therefore be understood as a system—implemented through multiple devices or tools—that generate data via sensors and biosensors. These data may support performance, health, or broader management within physical and sports activities.

### 5.2. Current Capabilities of Artificial Coaches

This section describes tools and applications and what artificial coaches can currently achieve through continuous or automated monitoring, based on current scientific evidence.

Regarding continuous monitoring and training-load management, devices can measure the trajectory of a person performing a sport action or manage routes according to greater feasibility. Other devices support menstrual-cycle management or quantify heart rate (HR) and heart rate variability (HRV), oxygen saturation, sleep and sleep quality, stress, and related variables.

Optical fiber sensing (OFS) has emerged as an efficient technology that, supported by advances in materials science (e.g., nanomaterials, flexible polymers, elastomers) and portable integration, offers high precision and electromagnetic immunity. It enables continuous monitoring in areas such as biomechanics and health, including physiological changes ranging from real-time fatigue metabolite monitoring to HR and respiratory metrics [77,78,79]. These capabilities are enabled by garments developed from the materials mentioned above [77], which transmit information to wearable devices or chips that can store these data [80]. Even so, further research on integrating AI and advanced biomaterials remains necessary [81].

When discussing automated systems, we refer to devices that can generate responses to user requests during interaction. Output depends on the parameter requested and may be directed to a coach or to an individual seeking support for physical or sport activity. In this context, yoga has been offered through metaverse environments, providing interactive experiences similar to those of an instructor by delivering personalized postural guidance based on real-time data analysis [82]. Systems can also be multimodal by incorporating baseline knowledge about the user, health-related issues such as diet, training objectives for coaches, or solutions intended to help a coach reach a defined goal (Creating an Artificial Coaching Engine for Multi-domain Conversational Coaches in eHealth Applications).

Deep learning is also presented as an automated, data-driven source of information, analyzing actions, objects, and classifications [83]. Beyond deep learning, combining data acquisition and processing has enabled proposals, for example, to improve movement technique in swimming [84].

Overall, evidence suggests that achieving sustained goal attainment and maintaining user engagement often requires long-term use of artificial-coach-oriented devices [85]. These objectives can be measured through artificial coaching implemented across multiple device types (garments, wearable devices, chips, etc.), providing answers related to health, training, or performance goals.

### 5.3. Evidence of Effectiveness of Artificial Coach With or Without Human Coaching

Interventions involving artificial coaches can lead to different interpretations, sometimes influenced by bias. To approach a more objective perspective, we analyze what the scientific evidence reports regarding the effectiveness of different exercise programs.

In the health domain, research was conducted to develop ‘E-supporter’, a tool designed as an artificial coach based on motivational messages, behavioural feedback and personalised support exercises, to overcome any apparent barriers that might exist within the set objectives. Fitbit Charge 2, a tracker bracelet, was used to monitor the activity. Physical activity, diet, self-efficacy, and stage of behavior change were assessed in individuals with type II diabetes, aiming to encourage healthier lifestyles. Results in a sample of 20 participants indicated positive perceptions and described E-supporter as useful and motivating; however, participants preferred using such systems combined with support from a healthcare professional [86].

Linking health and performance, Manescu’s big-data framework reported outcomes including a 12% reduction in hamstring injury rates in American football, a 16% improvement in key decision-making accuracy in basketball, and an 8% reduction in 100 m sprint times among athletes [66].

Virtual reality (VR), through immersion via headsets in digitally created environments, has shown positive results in physiological improvements comparable to conventional approaches, while also providing greater enjoyment, participation, and behavioral sustainability [87]. In older adults, optimized and personalized VR training (e.g., 30-min sessions) has been associated with reduced heart rate and glucose levels and highlights the effectiveness of personalization and continuous feedback [88]. In patients with heart failure, VR has been presented as a complementary approach that may improve performance and adherence in rehabilitation programs [89].

Within Physical Virtual Sports (PVS), such as indoor cycling or Just Dance, as the best-known activities, we also find digital football. The tool developed by Huszár and Ahikarla operates as a referee rather than an artificial coach; nonetheless, we understand that this example broadens the scope and has an impact on training planning by human coaches and the configuration of artificial coach systems [90] to ensure sustainability, efficiency, and fair play, reducing biases associated with doping or unethical activity.

Other evidence includes solutions using MediaPipe and a Multi-Layer Perceptron (MLP), a system focused on enabling users to improve their technique in basic exercises such as pull-ups, push-ups, or abdominal exercises through image-processing approaches [91]. In volleyball, similar cases have been reported through action recognition to assess performance and player behavior [92]. In Japan, a step-counting mHealth program that promoted the exchange of digital train tickets concluded that incentives encouraged active travel and increased public transport use [93]. Another study reported that technology can improve physical performance but not necessarily self-efficacy in amateur athletes [94]. Eye-tracking has been presented as a powerful tool to study behavioral and cognitive functioning in basketball and to inform training strategies to improve performance [95].

In performance contexts, laser-based technology has been described as an affordable option for evaluating linear sprint speed in outdoor team sports [96]. In basketball, machine-learning models have been proposed to evaluate tactical aspects in real time [97]. Finally, there are other sports, such as tennis, that also have monitoring systems, in this case, of forearm muscle fatigue—such as that developed by Ramírez et al., to distinguish fatigue vs. non-fatigue states, enabling training personalization and potential applications in rehabilitation or injury prevention [98].

Looking ahead, more research is needed on technological solutions already in use, as outcomes are not always positive when technology is integrated with physical activity [99]. Ensuring real-time accuracy and reliability of outputs generated by AI-based systems also remains essential [5]. Hybrid approaches that combine human and artificial coaches continue to be considered important [86,94]. Future trends in human–artificial coach interaction appear oriented toward training personalization, injury prevention, digital twin development, and talent identification [5]. Depending on how technology is applied and the surrounding legal framework, human involvement may be higher or lower, and in some cases, absent.

### 5.4. Why Are Elite Athletes Still Working with Human Coaches?

Given the current capabilities and possibilities of artificial coaches, it is important to address why elite athletes still work with human coaches. The emotional domain often appears central to this reasoning, together with ethical considerations and the experience and knowledge held by the human coach. This is what we intend to analyze in this section, as the pace of technological advancement reinforces the need to revisit these questions.

In decision-making and tactical knowledge, differences may still favor the human coach. In many cases, the AI “black box” and system traceability are not fully known, which can lead to limitations and generate disjointed or erroneous information. At the same time, technology can strengthen the human coach–athlete relationship by enabling new ways to structure conversations, deliver clear feedback, and reinforce defined goals [100]. Another reported factor is limited funding and accessibility. Thus, human coaches retain decision-making authority even when data are generated by AI, although strategic management may increasingly rely on AI-driven information as these systems mature [101]. Overall, research describes these tools as facilitators in their daily work rather than replacements for the human coach [100,101,102,103]. However, this is not the end of the matter, as it also suggests the need for training for those involved in the use of technology, legal regulations and associated data protection [104].

Emotion-detection systems have been explored in sport as a means to optimize performance [105]. In health and physical-activity contexts involving artificial coaches, evidence suggests that more authentic anthropomorphic design may strengthen parasocial relationships and promote user retention [106]. Economic factors also appear relevant in user loyalty and emotion detection within fitness platforms. It is true that outside the physical-sporting sphere, positive effects of artificial coaches have been reported in contexts such as healthcare and language learning [107,108,109]. Other studies highlight the importance of positive social relationships among agents involved in physical or sport activity [110] and call for deeper theoretical work that incorporates emotional factors [111]. Compassion and resilience are considered useful in difficult situations by technical staff [112], yet there is no evidence indicating that technology helps athletes determine compassion or increase resilience. Other studies describe human coaches as central to athlete preparation dynamics and as architects of the high-performance sport environment, not only a simple classic human coach [113]. Athletes also emphasize maintaining the human component in training [114]. Finally, several works highlight the need for human-centered practices and warn that excessive dependence on AI may undermine human aspects [115,116,117], including outside sport contexts [118].

Ultimately, any change requires critical analysis to evaluate the impact of implementation, clarify what is being externalized from human action to technology, and determine whether the solution is appropriate for the context. Performance and health settings differ, as do contexts involving children, adolescents, or adults. Return-to-play after injury, mental health considerations related to physical activity (performance or non-performance), and similar factors must be considered. In short, analysis and reflection should guide decisions, weighing objectives and required resources within a legal and ethical framework. Indeed, recent advances in large language models and reinforcement learning highlight that the capabilities of AI-based coaching systems should be understood as dynamic rather than static. LLMs can already generate context-sensitive, motivational, and empathetic responses, while also exhibiting limitations such as hallucinations, inconsistency, and lack of grounded responsibility [119,120]. As these systems continue to improve through iterative training, feedback, and human-in-the-loop refinement, certain informational, instructional, and communicative coaching functions may increasingly shift toward digital systems, whereas relational trust, ethical accountability, and responsibility for high-stakes decisions remain areas where human oversight is currently indispensable.

### 5.5. Key Limitations of the Artificial Coach

Various fields may present positive points or areas for improvement in terms of using an artificial coach to achieve their work objectives. Data handling and interpretability, potential bias, adherence to these systems, and the digital divide and accessibility are key factors when forming a positive, vigilant, or conservative assessment of artificial coach use. We exclude training from this analysis, as it is assumed that training is a prerequisite for the implementation and use of these systems.

In health contexts, adverse scenarios may emerge when patients misinterpret communications or do not perceive full support among stakeholders involved in communication in cases of obesity and mental health, resulting in insufficiently personalized outcomes [121]. Bias in AI, arising from training on specific datasets, reinforces the need for human-equivalent evaluation and for clear multidimensional validation frameworks [119,122]. This extends to issues of disclosure or privacy in coaching and the need to respond responsibly and appropriately to the high emotional burden that can arise in certain types of situations [123].

Despite promising technological progress, a gap remains between advances and practical application, as reliability and interpretability of sensor-based interventions still need improvement in athlete health contexts [81]. Legal responsibility must be considered in cases of technological failure, training overload due to malfunction, or misuse by an athlete or human coach [124]. The role of the human coach is also emphasized in programming effective training, for example, in personalized running supported by immersive and multimodal technologies [125]. In artistic gymnastics, qualitative evidence (45 interviews) highlights automation, mechanization, precision, and the system’s inability to evaluate artistry or provide human interaction [126].

GPS systems also involve variables that are not always transparent in how they contribute to final outputs. Therefore, it is recommended to include reporting satellite counts and device brands, disclosing updates or changes, using minimum time windows to identify efforts, applying caution with speed thresholds, anchoring speed zones to a single physiological metric, and interpreting accelerometer comparisons cautiously [127]. Although GPS use appears widely adopted among team-sport professionals [128], discrepancies remain in accelerometer use, and further consensus and research are required. Wearable devices also present limitations in privacy, cost, and accuracy, reinforcing the need for regulation and legal and privacy-related decision-making [75,129].

Finally, if universal access to these agents is pursued, broad population-level education is needed through organizations capable of reaching all groups. This should be supported by public institutions and non-profit organizations so that bias awareness and universal accessibility can be feasible, affordable, legitimate, and ethical.

## 6. Application Layers Built on Biosensor-Driven Adaptive Systems

Technological applications and tools related to musculoskeletal development and specific human physical capacities are in constant evolution. Large investment funds are increasingly viewing sport as a strategic economic sector, highlighting the societal impact of sport, physical activity, and exercise. The technologies discussed in this section, including digital twins, immersive environments, and virtual coaching, should be understood as application layers built upon biosensor-driven closed-loop systems. Their effectiveness, safety, and ethical viability are contingent upon upstream sensing accuracy, signal quality, and uncertainty-aware inference. Without validated biosensor inputs and clearly defined safety constraints, such technologies risk amplifying measurement error rather than enhancing training adaptation [6,11,13,81].

### 6.1. From Stand-Alone Wearable Devices to Intelligent Coaching Ecosystems

Technology has progressed from independent systems to integration across devices. This occurs through computing and measurement, with measurement increasingly embedded in wearable devices. Wearable devices now capture physiological parameters, location, and movement patterns [130]. While commonly implemented as wristbands or watches, integration into textiles and footwear is gaining traction, aiming for improved optimization [130].

More specifically, over the last decade, there has been a transition from wearable-device measurement of kinematic and cardiovascular responses in health and sport settings with heterogeneous reliability [40,131] to AI-enabled measurement within wearable devices using MLand statistical models to increase accuracy, reduce injury risk, and improve performance [132,133]. Reported outcomes of these integrations are positive, and unification of digital signal-processing pipelines is also progressing [134]. IoT integration has supported these processes at scale [135], and it is necessary to begin considering the emerging inclusion of wearable optical-fiber sensors [136].

Improved integration accuracy may benefit the general population, as increasing technological sophistication can contribute to cost reduction over time, improving accessibility across performance, health, and education contexts.

### 6.2. Athlete Digital Twins and Simulation-Based Coaching

Digital Twins (DT) are virtual duplicates of a physical asset created from multisensor data collected from that asset. They can be monitored in real time, enabling analysis and more effective and evidence-based decision-making [137]. Based on this premise, we outline several DT applications.

In sport, basketball is one of the most current examples. The study by Lv et al. combines DT technology and Multi-Agent Reinforcement Learning (MARL), integrating physical and virtual tactical simulation with real-time error correction and reporting positive results [30]. In athletics, DT-based approaches have been used as a preliminary step for high-performance training implementation, for example, triggering sprint starts when a virtual partner crossed a start signal marked on the ground, while analyzing body and visual movements [138]. DTs have also been considered in military training as valuable tools, although still in development [139].

Combining smart suits with DTs has been proposed for real-time human-body monitoring, with the aim of addressing limitations of other technologies; this is considered applicable to domains such as medicine and sport science [140]. Other DT-based monitoring approaches, such as upper-limb strength assessment and movement classification through 3D garment systems [141,142], may support advances in rehabilitation medicine and training.

Future DT development is expected to focus on data analytics, integration of AI or machine learning, and advanced sensing technologies to improve responsiveness and increase efficiency for end users [137], whether in performance or health contexts. Accuracy must be considered, as DTs must not only generate appropriate actions, but users must also adjust their movement correctly.

### 6.3. Immersive Coaching

Immersive training modalities are increasingly implemented through virtual environments using suits, gloves, and/or headsets, referred to as virtual reality (VR); through the overlay or visualization of real-world elements using augmented reality (AR) devices such as smartphones or tablets; and through mixed reality, which hybridizes both. The metaverse extends beyond localized VR environments toward broader virtual ecosystems, where, for example, multiple VR spaces may coexist. The concept of extended reality (XR) encompasses these non-analog modalities.

Regarding immersive technology within artificial coaching, applications have been reported across different contexts. Recent evidence highlights multidisciplinary AR practice. A program assessed the impact of AR-based multidisciplinary exercise on mood and salivary oxytocin; AR was delivered via avatars simulating self-paced cycling, producing significant results [143]. AR system design has also supported non-professional users in understanding movement and adjusting it via real-time feedback [144]. AR has also been applied to monitoring sport or educational activity, though less directly framed around the artificial coach concept [145,146].

VR has likewise shown emerging applicability across contexts. During the COVID-19 pandemic, VR was proposed as a solution to isolation by enabling exercise without attending a gym and without an on-site human coach [147]. VR has also been used for recreational or health-oriented users by providing 3D scenarios in gyms for indoor cycling [148], as support for coaches guiding dance learners in university contexts, and in table tennis and sailing as sport and educational modalities [149,150,151]. In basketball, which is one of the sports where research into these issues is being conducted, VR has been associated with improved psychological training attitude and positive effects on mental health and psychophysiological performance [152].

Finally, proposals have been described where an avatar functions as a session guide—an artificial coach—by providing real-time feedback during yoga practice [82]. AI-enhanced multimodal insole sensing systems (AEIS) have also been employed to enable such feedback loops. Commercial initiatives already exist that deliver fitness through the metaverse and XR. Across these developments, the protection of user data and the drafting of policies and ethical frameworks by governments remain essential to support responsible deployment [153].

### 6.4. The Rise of AI in Artificial Training

AI has become indispensable for increasing the effectiveness of the devices described in the previous section. Generative AI (GenAI) can produce training guidelines based on available scientific information and user-defined objectives, although domain knowledge remains necessary to apply them safely. This section provides examples of how AI is being integrated into training and the advances enabled in recent years.

Using reinforcement learning and motion capture, an artificial coach was proposed to estimate posture and build a training model, incorporating imitation rewards and penalties for incorrect patterns. Results were promising in terms of action accuracy, adaptation, and user satisfaction [154]. Lloyd et al. similarly reported in their review that AI-driven virtual assistants (i.e., artificial coaches) do not always produce positive effects in promoting physical activity, although several studies reported beneficial outcomes. They also noted that LLM-based chatbots provide richer, more flexible, and context-adapted experiences than simpler architectures, albeit with higher computational and energy costs [139]. Thus, despite accessibility and continuous feedback, such systems still show limitations related to redirecting certain activities as alternatives to specific tasks and to supporting interpersonal coach–athlete relationships.

As support tools, AI has been used to improve exercise accuracy or fitness-related outcomes in basketball [155], reduce injury risk in physical-education contexts through data-driven training plans [156], and support technical skill acquisition in tennis in combination with VR and wearable sensors [157]. A study in Germany by volunteer sports students (n = 212) in which artificial coach attributes were structured by sport students highlighted the need to align the artificial coach with the user’s psychological profile, particularly for achievement-related goals [158]. Emotional development remains a weak point, even when knowledge-related performance is robust across domains (health, performance, education, etc.).

Overall, the rapid integration of new hybrid training methodologies is creating new possibilities for human coaches, athletes, and physically active populations. Although the replacement of personal trainers by artificial coaches is already being discussed [137], rigorous systematization remains necessary before this can become a realistic pathway. In performance contexts, upcoming Olympic cycles may mark a turning point.

## 7. Ethical, Social, and Professional Implications

The integration of AI and ML into training and performance monitoring promises substantial gains in precision, personalization, and efficiency. However, these technological advances also introduce important ethical, social, and professional challenges [159,160]. The continuous collection of biological data, algorithmic decision-making, and the partial automation of coaching processes demand a critical evaluation of privacy, fairness, and the future role of human professionals. Ensuring responsible implementation requires a balance between technological innovation and the protection of athletes’ rights, health, and autonomy.

### 7.1. Data Privacy and Athlete Consent in Biosensing and AI Analytics

AI-driven coaching systems rely on large volumes of personal data generated from physiological, biochemical, biomechanical, and even psychological monitoring [161]. Although these datasets can support precise adaptation and performance optimization, they also raise concerns related to privacy, surveillance, and potential misuse of sensitive information [159].

Continuous biosensing and AI-based monitoring raise important concerns regarding informed consent and the potential shift from performance support to surveillance. Athletes may have a limited understanding of what data are collected, how they are processed, or who has access to them, particularly in professional or scholarship-dependent environments where monitoring may be perceived as obligatory rather than voluntary. In such contexts, consent risks become procedural rather than meaningful, and continuous data collection can undermine autonomy if not accompanied by clear governance, transparency, and revocable consent mechanisms.

In many cases, athletes may not fully understand what types of data are being collected, who has access to them, or how they are used. This poses challenges to meaningful informed consent, particularly in professional or scholarship-dependent environments where refusing monitoring could be perceived as detrimental to the athlete’s opportunities. Another emerging concern is the risk of re-identification—even anonymized physiological or motion-based data can frequently be traced back to a specific individual due to unique biological patterns [162].

To mitigate these risks, sport organizations and research institutions must ensure:

- clear and revocable informed consent,
- transparent policies on data storage, access, and deletion,
- use of data exclusively for health and performance-related purposes (not commercial exploitation),
- compliance with data protection frameworks, such as the GDPR or equivalent regional regulations.

Without such protections, digital training environments risk shifting from performance monitoring to invasive surveillance [163]. In many cases, athletes do not fully understand what types of data are being collected, who has access to those data, or how they will be used, which undermines the possibility of meaningful informed consent. Studies in elite sport have shown that athletes frequently sign consent forms without reading them in detail, and up to 74% report uncertainty about the destination of their physiological and biometric data once transferred to team or institutional servers [25]. This problem is particularly acute in scholarship-dependent or professional environments, where refusing biosensor monitoring may be perceived as a threat to playing time, selection, or contract renewal, effectively converting “consent” into a structural obligation [47]. An additional ethical concern is the growing evidence that anonymized biosensor data can be re-identified through unique physiological and biomechanical signatures. For example, de Montjoye et al. demonstrated that just four spatiotemporal points were sufficient to re-identify 95% of athletes in motion-tracking datasets, even after de-identification procedures [164], and Piciucco et al. found that individual users could be correctly re-identified with over 90% accuracy using heart rate variability and accelerometry profiles collected from consumer wearables [165]. These findings challenge the assumption that anonymization alone protects athlete privacy. Without rigorous safeguards, revocable consent, transparent data-management policies, secure storage, and compliance with data-protection regulations such as GDPR, digital monitoring risks shifting from performance optimization to a form of invasive surveillance, where athletes are continuously tracked, analyzed, and potentially evaluated beyond the original purpose of health or training [166].

### 7.2. Algorithmic Bias, Fairness, and Trustworthiness

ML models are only as fair as the data used to train them. When datasets are incomplete, unbalanced, or reflective of existing demographic inequalities, AI-based decision systems can generate biased outputs. In sport, this may lead to:

- inaccurate predictions of fatigue or injury in athletes whose physiological profiles differ from the majority,
- misclassification of women, youth, or athletes with atypical morphologies,
- reduction of opportunities due to erroneous risk profiling [167,168,169].

Limited algorithmic transparency (“black boxes”) further complicates accountability. If a model adjusts training load or flags a health risk, athletes and staff must be able to understand the rationale behind that decision. Emerging Explainable AI (XAI) approaches attempt to address this issue by designing more interpretable, auditable decision-making frameworks. However, algorithmic supervision, validation, and final judgment must remain human responsibilities [167,168].

### 7.3. The Professional Future of Coaches and Sport Scientists in an AI-Driven World

The increasing use of AI in coaching often triggers fears of professional displacement. Yet current evidence suggests that full automation is unlikely. AI excels at pattern recognition and quantitative optimization, but lacks qualities indispensable to human coaching:

i.   empathy,
ii.  motivational communication,
iii. contextual understanding,
iv.  leadership,
v.   and the ability to integrate psychological, social, and cultural variables [170].

The study by Barger et al. (2025) compared a 60-min coaching session delivered by a human coach versus a live-motion avatar presented as an AI coach. Results showed no significant differences in working alliance between conditions, with both groups reporting moderately high levels of connection, goal alignment, and perceived guidance. Qualitative feedback supported these findings, as participants described feeling heard and supported in both formats, and even expressed openness to developing a future coaching relationship with an AI-based coach. These results suggest that simulated AI coaching environments can elicit relational dynamics comparable to traditional human-led sessions [170]. Rather than replacing coaches, AI is reshaping their roles. Coaches are becoming data-literate decision-makers who combine technological insights with professional expertise. For instance, Zhu (2025) presents FASSLING, an AI-based life-coaching system that offers free, multilingual, 24/7 support, demonstrating improvements in accessibility, user engagement, and personalized interventions. This system emphasizes that AI should complement, rather than replace, human coaches due to ethical and emotional concerns [171]. Similarly, Haase (2025) reports that coaches currently use generative AI mainly for research, content development, and administrative tasks, with the human relationship remaining central to effective coaching. AI adoption is influenced by coaches’ technological literacy and attitudes, and persistent ethical issues reinforce the idea that GenAI is best positioned as an augmenting tool rather than a substitute for human expertise [172].

Similarly, sport scientists are shifting toward more analytical tasks, such as model validation, interpretation of multimodal metrics, and cross-disciplinary integration of physiological, biomechanical, and psychological data [158]. For instance, in Terblanche et al.’s longitudinal randomized-controlled trial comparing human coaching and an AI-chatbot coach over a ten-month period, both interventions significantly improved clients’ goal attainment compared to control groups. Unexpectedly, the AI-coach condition proved as effective as the human coach condition at the end of the trial, suggesting that AI-based coaching can match the efficacy of traditional human coaching under certain conditions [12].

In this sense, the future points toward human–AI collaboration, in which technology augments rather than replaces human expertise.

### 7.4. Ethical Guidelines for Responsible Implementation of AI in Human Training

The responsible use of AI in training environments requires a clear ethical framework that prioritizes athlete welfare above technological ambition. Central to this framework is the preservation of human oversight: AI systems should inform and support decision-making, but never function as autonomous authorities capable of determining training loads or medical risks without expert supervision [173,174]. Ensuring transparency and interpretability is equally essential. Coaches, sport scientists, and athletes must be able to understand how models generate their recommendations, particularly when these outputs influence health, performance, or professional opportunities [175]. Carrió Sampedro (2023) argues that the rapid adoption of AI in sport is advancing faster than the ethical and regulatory frameworks needed to protect athletes’ rights and wellbeing. The report warns that biometric monitoring, performance prediction, and automated training decisions can threaten autonomy, dignity, and health if deployed without strong safeguards. It concludes that sports organisations, especially the Olympic movement, must develop clear governance, legal accountability, and athlete-centred ethical standards before AI becomes structurally embedded in high-performance systems [176].

Data protection represents another pillar of ethical implementation. Biosensing and multimodal monitoring technologies generate highly sensitive information, which should be collected with parsimony, stored securely, and anonymized whenever possible [177]. Access must be restricted to qualified personnel, and the athlete should retain the right to withdraw consent or request data deletion without negative consequences. Fairness is also indispensable: AI systems must be validated across diverse samples to prevent discriminatory outcomes based on sex, age, physiology, or competitive level. This requires continuous auditing, since algorithmic bias can emerge over time as new data reshape model behaviour [178].

Beyond technical safeguards, responsible implementation also demands proportionality and accountability. AI should only be applied when it offers a demonstrable benefit that surpasses traditional monitoring methods, and any potential harm derived from faulty predictions or misinterpretation must be attributable to the organization and professionals deploying the system. Ultimately, an ethical approach to AI in human training recognizes that performance enhancement can never come at the expense of autonomy, dignity, or fairness. By ensuring transparency, protecting privacy, supporting human-centred decision-making, and actively mitigating bias, AI can evolve as a trustworthy and socially responsible tool within the future of sport.

## 8. Challenges and Future Directions

Although AI-driven coaching systems have progressed rapidly, several technical, scientific, and ethical challenges remain before fully autonomous coaching can be considered viable. Future innovation will require not only better algorithms and biosensing hardware, but also stronger scientific validation, interdisciplinary collaboration, and a paradigm shift in how coaching roles are understood in the intelligent era [179].

### 8.1. Standardization and Validation of AI-Driven Biosensor Systems

A persistent limitation of current AI-based coaching platforms is the absence of standardized protocols for biosensor calibration, data acquisition, and algorithmic evaluation. Wearables often differ in sampling frequency, signal quality, and noise resistance, making cross-study comparisons difficult and limiting clinical validity. Most AI training models are highly sensitive to data quality; inaccurate physiological or biomechanical inputs can generate misleading recommendations or risk-amplifying errors. In fact, recent evidence shows that while smartphone-based biosensing has advanced to real-time, low-cost diagnostic prototypes with high sensitivity, its broader deployment is still hindered by calibration inconsistencies, interoperability problems, manufacturing constraints, and limited long-term validation [180].

### 8.2. The Need for Interdisciplinary Integration (AI, Physiology, Psychology)

Training adaptation is inherently biopsychosocial: physiological readiness, psychological state, sleep, motivation, stress, and environmental context all co-determine performance. Yet the majority of current AI models rely primarily on physiological or biomechanical data, with minimal integration of cognitive, emotional, or behavioral indicators [181,182]. This reductionist approach limits ecological validity and may produce overly mechanical or context-blind prescriptions.

The next generation of systems must merge:

- AI and ML (pattern recognition, prediction, adaptive control),
- physiology and biomechanics (fatigue, workload, injury risk),
- psychology and behavioral science (motivation, adherence, perceived effort, decision-making).

Such integration would allow AI not only to monitor the body but to interpret the athlete as a complex, dynamic human agent, bridging quantitative precision with behavioral understanding.

### 8.3. Human–Machine Synergy as the Optimal Paradigm

Current evidence increasingly supports a hybrid model: AI excels at large-scale analytics and precise workload modulation, while human coaches retain superiority in empathy, contextual judgment, leadership, and emotional regulation. Instead of asking whether AI can replace the human coach, the more relevant question is how technology and expertise can complement one another.

In practice, this synergy could include:

- real-time predictive analytics guiding coach decisions,
- automated detection of risk states (fatigue, dehydration, technique degradation),
- coach-mediated interpretation of algorithmic recommendations,
- continuous learning from both human expertise and sensor-based feedback.

Such models position AI not as a competitor, but as a powerful decision-support system that enhances human capability. For instance, some research already illustrates this integrative approach and combined physiological signals and psychological metrics in a machine-learning model that improved performance prediction accuracy [183]. Biró et al. applied AI to IMU-based multivariate data to predict fatigue and stamina in real time, enhancing adaptive training control [184]. Ayala et al. used machine-learning algorithms to identify early injury risks in athletes with around 90% accuracy, offering actionable insights for prevention [185].

### 8.4. Beyond Replacement: Reimagining the Coach’s Role in the Intelligent Era

The intelligent era does not eliminate the need for coaches; it transforms what coaching means. As automation expands, coaches are shifting toward roles centered on strategic decision-making, psychological support, ethical oversight, and the interpretation of complex data. AI takes responsibility for monitoring, optimization, and pattern recognition; humans focus on meaning, motivation, and individualized care.

**Figure 2:**
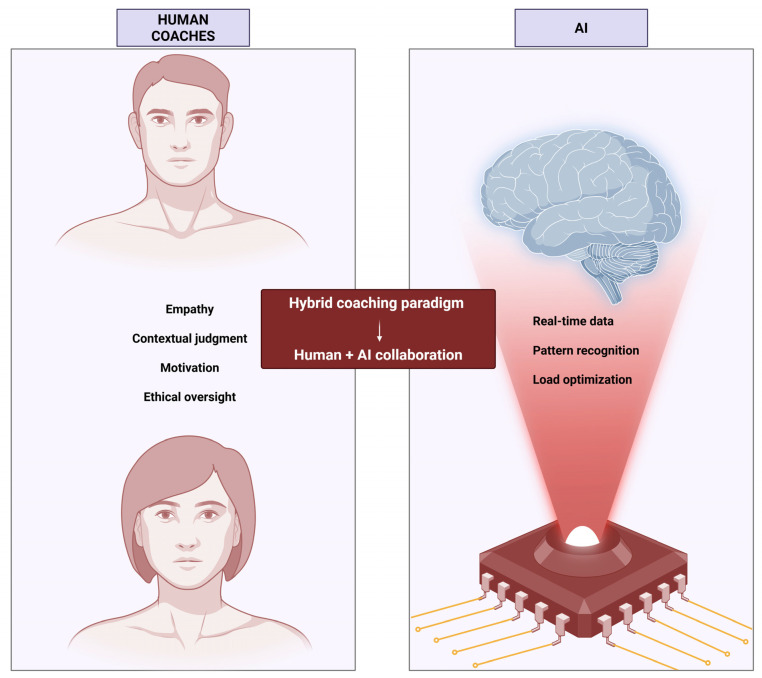
Human and AI Coaches: Complementary Competencies in Training Optimization.

Such evidence supports the idea that AI does not eliminate the role of the coach but transforms it, shifting human expertise toward strategy [186], contextual judgement and motivational support, while automation handles monitoring, optimization and pattern recognition. Studies with AI-avatar coaches even show similar working alliance and user acceptance compared to human coaches [170,187], although relational and ethical dimensions remain uniquely human. Consequently, the future points toward augmented coaching supported by digital literacy and human-AI collaboration rather than professional replacement (Figure 2).

Emerging evidence including studies showing high user acceptance of avatar-based coaches and AI-driven platforms suggests that digital systems can match or even exceed human performance on certain tasks. However, relational, ethical, and contextual dimensions remain uniquely human. Thus, rather than being replaced, coaches become augmented professionals whose work is amplified by intelligent tools. In the longitudinal study Comparing AI and Human Coaching Goal Attainment Efficacy [12], participants who worked with either a human coach or an AI-based coaching chatbot achieved significantly higher goal-attainment scores than control groups, and by the ten-month follow-up, the AI coach was as effective as the human coach, with both producing large and comparable effects on goal progress. Similarly, Arakawa et al.’s pilot study showed that a blended model combining a large-language-model chatbot with a human coach supported self-reflection and leadership development, with users highlighting the chatbot’s accessibility and reasoning support while noting that deeper emotional and introspective dialogue still depended on human interaction [188].

Thus, the future of coaching will depend not on substitution, but on re-training, digital literacy, and a collaborative human–AI ecosystem. In this context, ongoing technological convergence is enabling emerging application layers such as digital twins, immersive environments, and AI-enhanced coaching platforms, which should be understood as downstream implementations built upon biosensor-driven adaptive training architectures. Their effectiveness remains contingent on sensor validity, model robustness, and human oversight. Table 4 summarizes representative examples of these developments.

## 9. Conclusions

AI is reshaping the foundations of training by enabling continuous biosensing, multimodal data integration, and predictive modelling capable of adjusting workloads with unprecedented precision. These systems have demonstrated clear strengths in monitoring, pattern recognition, risk detection, and the automation of decision-support processes. Emerging technologies such as digital twins, immersive environments, and generative AI further expand the potential for highly personalized, adaptive, and scalable training ecosystems.

However, the evidence reviewed also reveals significant boundaries. AI systems remain constrained by sensor accuracy, calibration inconsistencies, algorithmic bias, and a limited capacity to interpret psychological, contextual, and environmental variables. More importantly, they lack the relational, emotional, and ethical competencies that underpin motivation, adherence, learning, and athlete wellbeing. The coach’s role is not only to prescribe training loads but to create meaning, trust, and a supportive climate that guides individuals through uncertainty, effort, and personal growth, dimensions that current AI cannot reproduce.

The future of coaching is not defined by substitution but by intelligent integration. AI should be understood as an augmenting layer that enhances human expertise, offering precision and scalability while the coach provides contextual judgment, leadership, and ethical oversight. Advancing this synergy requires interdisciplinary collaboration, rigorous validation standards, transparent governance, and the development of digitally literate professionals. Only through this balanced approach can AI contribute responsibly and effectively to the evolution of sport and exercise training. Accordingly, its use in coaching contexts should not be interpreted as a replacement of coaching expertise, but as a constrained decision-support layer whose outputs require validation, uncertainty awareness, and professional oversight. Without rigorous validation, bias control, and human accountability, AI-driven systems risk amplifying errors rather than improving training quality or athlete safety.

## Figures and Tables

**Table 1:** Evidence map of core empirical studies informing AI-driven biosensor-based adaptive training systems.

Study	Modality	Cohort Size	Setting	Outcome/Label	Model Type	Evaluation Protocol	Key Findings
Santos-García et al., 2022 [15]	HRV (PPG/ECG)	Elite female soccer players (n = 8)	Field	Internal load, fatigue	Statistical/ML regression	Athlete-wise, longitudinal	HRV reliably tracked internal load fluctuations during competitive microcycles
O’Connor et al., 2024 [16]	HRV (PPG)	Pro Australian football players (n = 46)	Field	Load–recovery dynamics	Time-series models	Longitudinal, season-wise	Waking HRV tracked cumulative load across two seasons
Elfouly & Alouani, 2025 [17]	EMG	Resistance-trained adults	Lab	Neuromuscular fatigue	ML pattern recognition	Within-session validation	EMG-derived features detected fatigue onset
Luo et al., 2025 [18]	Sweat electrolytes	Trained cyclists (n = 55)	Field	Electrolyte balance	Signal processing + ML	Trend validation	Sodium/potassium dynamics reflected metabolic stress
Yang et al., 2024 [19]	Sweat lactate sensor	Endurance athletes (NR)	Lab + Field	Metabolic threshold	Regression	Concurrent validity vs. blood lactate	Wearable sweat lactate strongly correlated with blood lactate (r > 0.85)
Swetha et al., 2022 [20]	Salivary cortisol	Healthy adults	Lab	Stress response	Electrochemical sensing + thresholds	Concurrent validation	Non-invasive cortisol tracking during exercise-induced stress
Alzahrani & Ullah, 2024 [21]	IMU (acc/gyro)	Mixed-sport athletes (n = 50)	Field	Workload, technique	ML classifiers	Cross-validation	IMU features captured fatigue-related mechanical changes
Zhang et al., 2025 [22]	IMU motion signals	Simulated + real data	Lab	Motion reconstruction	Kalman + ML hybrid	Signal-level validation	AI filtering improved trajectory accuracy
Wen & Diliya, 2024 [23]	Multisensor fusion	Recreational athletes (n = 500)	Field	Training personalization	Variational autoencoder	Latent feature analysis	Latent patterns captured individual adaptation
Wang et al., 2025 [24]	Multimodal (HRV + IMU)	Competitive tennis athletes (n = 100)	Field	Training adaptation	Supervised ML	RCT, group comparison	Wearable-guided training improved HRV recovery and performance stability
Lundstrom et al., 2024 [25]	HR, VO_2_ estimation	Elite swimmers (n = 23)	Field	Energy expenditure	Regression ML	Criterion validity	Wearable metrics correlated strongly with training load
Rothschild et al., 2024 [26]	HRV-based recovery	Endurance athletes (n = 43)	Field	Next-day readiness	ML regression	Prospective validation	ML reduced prediction error vs. baseline models
Qin et al., 2025 [27]	Multimodal (HRV, VO_2_, EMG)	Mixed athletes (n = 480)	Lab + Field	Performance	Deep learning	Train–test split	Multimodal DL explained 90% of performance variance
Guneralp et al., 2025 [28]	GPS + workload	Team-sport athletes (n = 60)	Field	Training adaptation	XGBoost	Cross-validation	ML differentiated adaptation patterns across modalities
Silacci et al., 2024 [29]	Wearable physiology	Cyclists (n = 6)	Field	Recovery readiness	ML decision system	Pilot deployment	Overestimation of readiness when psychosocial data absent
Lv et al., 2024 [30]	Digital twin + sensors	Basketball players (n = 120)	Lab + Field	Tactical performance	MARL	Simulation + real feedback	Digital twin supported adaptive tactical decisions

**Table 2:** Biosensors used in AI-driven training systems: measured signals, training applications and validation evidence.

Sensor/Technology	Primary Signals Measured	Main Training Applications	Validation/Evidence (From Included Studies)
HR & HRV wearable monitoring (PPG/ECG)	Heart rate, HRV, autonomic balance	Internal load tracking, recovery profiling, fatigue detection	HRV reflects internal load and high-intensity running in elite female soccer players [15]; strong correlations between HRV indices and training intensity in resistance exercise [40]; long-term association between waking HRV and training load over two competitive seasons [16]
Biochemical sweat sensors	Lactate, electrolytes, metabolic markers	Metabolic threshold detection, hydration monitoring, metabolic fatigue	Continuous sweat lactate monitoring correlates strongly with blood lactate [19]; sodium/potassium microfluidic patch reflects electrolyte dynamics and metabolic stress in cyclists [18].
Salivary biochemical sensors	Cortisol	Stress monitoring, recovery state	Electrochemical wearable sensor detects cortisol fluctuations linked to exercise-induced stress [20]
IMU-based biomechanical tracking	Acceleration, angular velocity, movement variability	Technique assessment, workload estimation, fatigue-related mechanics	Advanced biomechanical analytics support precision monitoring in sports performance [21]
EMG wearables	Muscle activation, neuromuscular fatigue	Strength training monitoring, motor control, fatigue thresholds	EMG applied to neuromuscular and fatigue assessment during exercise [17]
Combining multimodal biosensors with AI	Physiological + biomechanical + stress data	Real-time adaptive feedback, personalized load control	12-week controlled study: continuous wearable feedback improved HRV recovery, reduced perceived fatigue, and enhanced stability in performance [24]; wearable physiological metrics correlate with training load and energy expenditure in elite swimmers [25]

Note. HR = heart rate; HRV = heart rate variability; PPG = photoplethysmography; ECG = electrocardiography; IMU = inertial measurement unit; EMG = electromyography; ML = machine learning.

**Table 3:** Recommended preprocessing and QC pipeline by sensing modality (typical ranges).

Modality	Typical Sampling Rate	Preprocessing (Minimum)	Artifact Detection/QC	Synchronization/Latency Notes	Recommended Windowing
PPG (wrist)	25–200 Hz	band-pass + detrend; motion-compensation	SQI/confidence index; accel-gated rejection	align to IMU timestamps; account for PPG sensor latency (device-dependent)	8–30 s windows; 50% overlap (HR/HRV use longer)
ECG (chest)	250–1000 Hz	band-pass; R-peak detection	beat-quality flags; ectopic removal	ECG often time reference; align other streams to ECG	10–60 s for HRV; shorter for HR
EMG	1000–2000 Hz	band-pass + notch; rectification; RMS/envelope	electrode pop/noise detection; SNR threshold	align to IMU/video; watch buffering delays in wearables	100–250 ms for activation; 250–1000 ms for classification
IMU (acc/gyro)	50–500 Hz	gravity removal; drift correction; smoothing	saturation checks; plausibility bounds	timestamp unification; resample to common grid	1–5 s; 50% overlap (technique), shorter for events
Sweat (electrochemical)	0.1–1 Hz (or per sample)	temperature compensation; baseline correction	sensor drift/outlier rules; flow/sweat-rate gating	slow dynamics; do not align to high-freq by naive interpolation	1–10 min windows (trend-focused)

Notes: PPG, photoplethysmography; ECG, electrocardiography; EMG, surface electromyography; IMU, inertial measurement unit; HR, heart rate; HRV, heart rate variability; SQI, signal quality index; SNR, signal-to-noise ratio; MVC, maximum voluntary contraction; QC, quality control.

**Table 4:** Summary of emerging technologies in artificial coaching.

Type of Technology	Context of Application	Reference
Wearables	Unification of the digital signal process	Procházka & Charvátová, 2025 [134]
Internet of Things, Sensors, Wearables and AI	Integration into sensors and wearable devices to provide real-time feedback and data analysis	Azhagumurugan et al., 2025 [135]
Optical Fibre, Wearables, Internet of Things and AI	Real-time monitoring of posture, movement, vital signs, physiological parameters, and activity tracking	Hou et al., 2026 [136]
Digital Twins	Use of Digital Twins in athletics	Chomienne et al., 2024 [138]
Digital Twins	Military training	Lloyd et al., 2023 [139]
Digital Twins	Classification of movement type	Jiang et al., 2024 [141]; Guo et al., 2024 [142]
Extended Reality and Augmented Reality	Cyclo training coach	Shima et al., 2024 [143]
Augmented Reality	Real-time feedback	Wu et al., 2023 [144]
Augmented Reality	Monitoring physical activity in education and health settings	Zhu et al., 2025 [145]; Omarov et al., 2024 [146]
Virtual Reality	Virtual environment with artificial coach	Peng et al., 2022 [147]
Virtual Reality	Cyclo training coach	Ali Akhtar et al., 2022 [148]
Virtual Reality	Artificial coach assists human coach in sailing, table tennis and dance	Zhao et al. 2025, Ji et al. 2023, Polechoński, 2024 [149,150,151]
Virtual Reality	Feedback as an artificial coach in yoga	Wang et al., 2025 [82]
Deep Learning and Motion Capture Technology	Real-time feedback, training planning, reward and penalty system	Ma & Meng, 2025 [154]
AI	Improving physical fitness in basketball. Combination of artificial coach and human coach in basketball	R. Liu & Shen, 2025 [155]
AI	Physical Education and injury prevention	Sun, 2025 [156]
AI and Virtual Reality	Learning technical skills in tennis	S. Liu et al., 2024 [157]

## Data Availability

No new data were created or analyzed in this study. Data sharing is not applicable.
